# Fibrinogen-to-Platelet Ratio and Hematologic Inflammatory Indexes in Spondylarthritis

**DOI:** 10.3390/jcm15082926

**Published:** 2026-04-12

**Authors:** Roxana Doina Ungureanu, Cristina Elena Bita, Mirela Nicoleta Voicu, Adina Turcu-Stiolica, Sineta Cristina Firulescu, Mihai Turcu-Stiolica, Andreea Lili Bărbulescu, Stefan Cristian Dinescu, Florentin Ananu Vreju

**Affiliations:** 1 Doctoral School, University of Medicine and Pharmacy of Craiova, 200349 Craiova, Romania; roxanadoinaungureanu@gmail.com; 2 Department of Rheumatology, Faculty of Medicine, University of Medicine and Pharmacy of Craiova, 200349 Craiova, Romania; cristina.gofita@umfcv.ro (C.E.B.); sineta.firulescu@gmail.com (S.C.F.); stefan.dinescu@umfcv.ro (S.C.D.); florentin.vreju@umfcv.ro (F.A.V.); 3 Discipline of Clinical Pharmacology, Faculty of Medicine, Carol Davila University of Medicine and Pharmacy, 020021 Bucharest, Romania; 4 Biostatistics Department, Faculty of Pharmacy, University of Medicine and Pharmacy of Craiova, 200349 Craiova, Romania; adina.turcu@umfcv.ro; 5 Health Economics and Outcomes Research Department, Faculty of Medicine, “Iuliu Haţieganu” University of Medicine and Pharmacy Cluj-Napoca, Victor Babes Street No. 8, 400012 Cluj-Napoca, Romania; 6 Rheumatology Department, Emergency County Clinical Hospital of Craiova, 1 Tabaci Street, 200642 Craiova, Romania; mihaiturcu0812@gmail.com; 7 Department of Pharmacology, Faculty of Medicine, University of Medicine and Pharmacy of Craiova, 200349 Craiova, Romania; andreea.barbulescu@umfcv.ro

**Keywords:** spondylarthritis, psoriatic arthritis, fibrinogen-to-platelet ratio, biomarkers, disease activity, BASDAI, principal component analysis, ROC curve, inflammatory markers

## Abstract

**Background/Objectives**: Spondylarthritis (SA) is characterized by high clinical heterogeneity, often resulting in a discrepancy between systemic inflammation and patient-reported symptoms. While hematologic indices are emerging as cost-effective biomarkers, their role in phenotypic differentiation remains unclear. We investigated the utility of traditional inflammatory markers, including the novel fibrinogen-to-platelet ratio (FPR), in differentiating SA subtypes and predicting patient-reported disease activity. **Methods**: This cross-sectional study included 64 patients with spondylarthritis: axial SA (*n* = 32), peripheral SA (*n* = 8), and psoriatic SA (*n* = 24). Clinical assessments included the Bath Ankylosing Spondylitis Disease Activity Index (BASDAI) and Functional Index (BASFI). Systemic inflammation was evaluated via C-reactive protein (CRP), fibrinogen, and calculated ratios (NLR, PLR, MLR, and FPR). Principal Component Analysis (PCA) was employed to map the inflammatory architecture, while Receiver Operating Characteristic (ROC) curves evaluated the predictive power for high disease activity (BASDAI ≥ 4). **Results**: Significant phenotypic differences were observed with the FPR demonstrating superior discriminative capacity (*p* = 0.003). Patients with peripheral SA exhibited significantly higher FPR (median 1.88) compared to axial (1.33) and psoriatic (1.32) subtypes, and the dedicated ROC analysis for phenotypic discrimination yielded an AUC of 0.866 (95% CI: 0.745–0.987) (1.36, *p* = 0.039). HLA-B27 prevalence was low overall (31.3%) and in psoriatic SA (4.2%, *p* = 0.012). Correlation analysis revealed strong associations between BASDAI and BASFI (ρ = 0.79), NLR and MLR (ρ = 0.78), and CRP and fibrinogen (ρ = 0.66). PCA identified two independent inflammatory dimensions explaining 74.8% of variance: neutrophil-hypercoagulable axis (41.4%, driven by NLR, PLR, and MLR), and an acute-phase/fibrinogen axis (33.4%, driven by CRP, fibrinogen, and FPR). Notably, FPR clustered with acute-phase reactants rather than leukocyte-derived ratios, supporting its role as a marker of systemic inflammatory burden. Although fibrinogen is involved in the coagulation cascade, the absence of direct coagulation markers precludes definitive characterization of this component as hypercoagulable. ROC analysis revealed that fibrinogen showed the highest discriminative ability for disease activity (BASDAI ≥ 4), with an AUC of 0.690 (95% CI: 0.519–0.861), followed by NLR (0.621), MLR (0.592), and FPR (0.583). However, overall discriminative performance remained modest, with most 95% confidence intervals crossing 0.5. **Conclusions**: FPR emerges as a robust phenotypic biomarker capable of discriminating against SA subtypes, particularly identifying peripheral SA with high accuracy and excellent negative predictive value. In contrast, its ability to predict patient-reported disease activity remains limited, reinforcing the distinction between trait and state biomarkers. Exploratory PCA revealed that FPR clusters with acute-phase reactants rather than leukocyte-derived ratios, supporting its biological link to systemic inflammatory burden. These findings advocate for a dual-purpose biomarker approach in SA: FPR for phenotypic stratification and fibrinogen for activity assessment, while clinical indices remain indispensable for symptom monitoring. Validation in larger, prospective cohorts is warranted.

## 1. Introduction

Spondyloarthritis (SA) is a heterogeneous group of chronic inflammatory rheumatic diseases that include axial and peripheral forms, characterized by enthesitis, sacroiliitis, and synovitis, with varying degrees of structural damage [1,2]. Delayed diagnosis remains common, especially in axial disease, leading to prolonged untreated inflammation and irreversible structural progression [3,4]. Despite advances in biologic therapies targeting TNF and the IL-17 axis, a considerable proportion of patients continue to exhibit persistent symptoms, radiographic progression, or treatment refractoriness, underscoring the ongoing unmet clinical need in this population [5].

SA is characterized by heterogeneous inflammatory pathways and variable systemic expression, which has sparked interest in identifying accessible biomarkers that reflect disease activity and predict long-term outcomes. Traditional acute-phase reactants such as C-reactive protein (CRP) and erythrocyte sedimentation rate (ESR) remain the most widely used laboratory markers, yet their sensitivity is limited, particularly in axial disease, where a substantial proportion of patients exhibit normal values despite active inflammation [6,7]. Consequently, attention has shifted toward hematologic indexes derived from complete blood counts, including the neutrophil-to-lymphocyte ratio (NLR), platelet-to-lymphocyte ratio (PLR), monocyte-to-lymphocyte ratio (MLR), and composite indices such as the systemic immune-inflammation index (SII) [8]. These markers are inexpensive, reproducible, and reflect systemic inflammatory and thrombo-inflammatory responses; however, their prognostic utility in SA remains inconsistent [9]. While some studies suggest modest associations with disease activity scores or radiographic progression, the predominantly compartmentalized and IL-17/IL-23-driven inflammation typical of SA may limit the sensitivity of peripheral blood–derived markers [1,5].

The fibrinogen-to-platelet ratio (FPR) has recently emerged as a composite inflammatory marker that integrates the acute-phase response and thrombo-inflammatory activity [10]. Fibrinogen, a hepatic acute-phase protein primarily induced by IL-6, reflects systemic inflammatory burden, while platelet count is influenced by cytokine-driven megakaryopoiesis and inflammatory activation [11]. In SA, where systemic inflammation varies between axial and peripheral phenotypes, FPR may provide additional insight beyond conventional leukocyte-derived ratios.

The unifying hypothesis of this study is that systemic hematologic inflammatory markers capture distinct and complementary dimensions of inflammation in SA, which may serve different clinical purposes: phenotypic stratification versus disease activity monitoring. Specifically, we pursued three objectives: (1) to determine whether hematologic inflammatory indices, particularly the novel FPR, can discriminate between axial, peripheral, and psoriatic SA phenotypes; (2) to characterize the underlying inflammatory architecture of SA through exploratory PCA, identifying independent dimensions of systemic inflammation; and (3) to evaluate whether these markers can predict patient-reported disease activity (BASDAI ≥ 4), thereby testing the assumption that systemic inflammatory burden directly translates into clinical symptoms.

## 2. Materials and Methods

### 2.1. Methodology

This cross-sectional study enrolled 64 consecutive patients with SA who were treated at the Department of Rheumatology of the University of Medicine and Pharmacy of Craiova, Romania. Of these, 32 patients had predominantly axial involvement (axSA), 8 had predominantly peripheral SA (pSA), and 24 were diagnosed with psoriatic arthritis (PsA). Patients with ankylosing spondylitis fulfilled the 1984 modified New York classification criteria, while those with psoriatic arthritis met the 2006 CASPAR classification criteria. Classification into axial or peripheral phenotypes was based on the predominant pattern of clinical involvement (sacroiliitis and spinal disease for axSA; peripheral arthritis, enthesitis, or dactylitis for pSA).

This study was conducted in accordance with the Declaration of Helsinki and was approved by the Ethical Committee of the University of Medicine and Pharmacy of Craiova, Romania (approval number 201, 24 November 2021). All patients provided written informed consent prior to inclusion in this study.

### 2.2. Samples

Blood samples were collected after an overnight fast. Laboratory assessments performed in all patients included erythrocyte sedimentation rate (ESR), C-reactive protein (CRP), complete blood count (CBC), and fibrinogen levels. Samples were analyzed using the ADVIA 2120i automated hematology analyzer (Siemens, Munich, Germany).

Inflammatory indices were calculated as follows: NLR (neutrophil-to-lymphocyte ratio) was determined by dividing the absolute neutrophil count by the lymphocyte count; MLR (monocyte-to-lymphocyte ratio) by dividing the monocyte count by the lymphocyte count; PLR (platelet-to-lymphocyte ratio) by dividing the platelet count by the lymphocyte count; and FPR (fibrinogen-to-platelet ratio) by dividing the fibrinogen level by the platelet count.

### 2.3. Statistical Analysis

We assessed the normality of the continuous variables (age, BMI, CRP, NLR, PLR, MLR, BASDAI, BASFI, fibrinogen, fibrinogen/platelets ratio) using the Shapiro–Wilk test. Data are presented as mean ± standard deviation and median with interquartile range (IQR). Categorical variables are expressed as frequencies and percentages. To verify differences among the three types of spondyloarthritis (Axial SA, Peripheral SA, and Psoriatic SA), we used the Kruskal–Wallis test, followed by Dunn’s test or the Mann–Whitney U test with Bonferroni correction for pairwise comparisons. For categorical variables (sex, diabetes, HLAB27), we used Fisher’s exact test when expected cell counts are <5 (likely needed given your small sample sizes, especially for Peripheral SA with n = 8). Spearman correlation coefficients were calculated to assess relationships between demographic, clinical, and laboratory variables, with correlation strength interpreted as weak (ρ < 0.3), moderate (0.3 ≤ ρ< 0.7), or strong (ρ ≥ 0.7). Principal component analysis (PCA) with varimax rotation was conducted on inflammatory markers (CRP, NLR, PLR, MLR, fibrinogen, and FPR) to identify underlying patterns of inflammation. The Kaiser–Meyer–Olkin (KMO) measure of sampling adequacy and Bartlett’s Test of Sphericity were used to assess the suitability of the data for PCA. Components with eigenvalues > 1.0 were retained, and the scree plot was examined to confirm the optimal number of components. All statistical analyses were performed using R software (Version 4.1, ggplot2, ppcor, factoextra, psych packages). The significance level was set at *p* < 0.05. For multiple pairwise comparisons, we applied the Bonferroni correction (*p* < 0.017 for 3 comparisons).

## 3. Results

### 3.1. Baseline Characteristics and Comparison Between Spondylarthritis Subtypes

Table 1 presents the baseline characteristics of the 64 patients with spondylarthritis (SA), stratified by disease subtype: axial SA (*n* = 32), peripheral SA (*n* = 8), and psoriatic SA (*n* = 24). Significant differences were observed in age distribution among the three groups (*p* < 0.001). Patients with peripheral SA were younger (median 42.5 years, IQR 37–45.8) compared to those with axial SA (median 47.5 years, IQR 43–55.3) and psoriatic SA (median 59.5 years, IQR 53–70.5), with psoriatic SA patients being the oldest.

Sex distribution showed significant difference between groups (*p* = 0.010), with males representing 43.8% of the total cohort, most of them with axial SA (31.2%). BMI did not differ significantly among the three subtypes (*p* = 0.113), although psoriatic SA patients showed numerically higher median BMI values (30.3 kg/m^2^, IQR 25.5–32.1).

Diabetes prevalence differed significantly between groups (*p* < 0.001), with rates of 9.4%, 12.5%, and 12.5% in axial, peripheral, and psoriatic SA, respectively. HLA-B27 positivity also showed significant differences (*p* = 0.012), being most prevalent in axial SA (12.5%) and peripheral SA (37.5%), while absent in the psoriatic SA group.

Inflammatory markers, including CRP (*p* = 0.75), NLR (*p* = 0.474), PLR (*p* = 0.374), and MLR (*p* = 0.764), showed no significant differences among the three SA subtypes, despite slight differences in mean values. Disease activity and functional indices, as measured by BASDAI (*p* = 0.247) and BASFI (*p* = 0.233), were comparable across groups.

Fibrinogen levels showed a significant overall difference between groups (*p* = 0.037). Post hoc pairwise comparisons revealed that peripheral SA patients had significantly higher fibrinogen levels (median 452 mg/dL, IQR 439–522) compared to psoriatic SA patients (median 361 mg/dL, IQR 312–427; *p* = 0.037), as the difference between axial and peripheral SA approached the same statistical significance (*p* = 0.04).

The fibrinogen-to-platelet ratio differed significantly between groups (*p* = 0.003). Pairwise comparisons demonstrated that peripheral SA patients had significantly higher ratios (median 1.88, IQR 1.62–2.21) compared to axial SA patients (median 1.33, IQR 1.09–1.44; *p* = 0.002) and psoriatic SA patients (median 1.32, IQR 1.22–1.51; *p* = 0.03).

### 3.2. Correlations Between Demographic, Clinical, and Laboratory Variables

**Figure 1:**
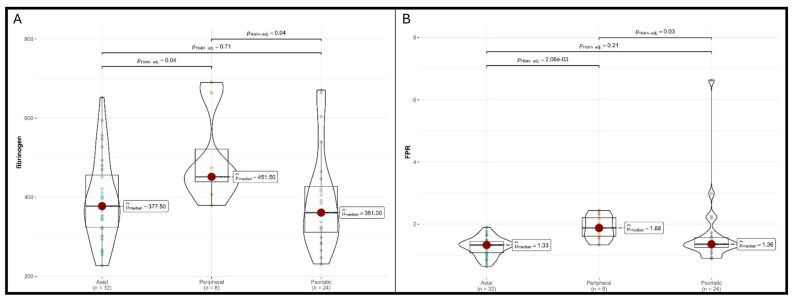
Comparison of clinical characteristics by diagnostic. (**A**) Fibrinogen. (**B**) Fibrinogen-to-platelet ratio (FPR).

Spearman’s rank correlation analysis was performed to examine the relationships among demographic, clinical, and laboratory variables in the entire cohort of 64 patients with spondylarthritis, as shown in Figure 1.

Strong positive correlations were identified between BASDAI and BASFI (ρ = 0.79, *p* < 0.001), indicating close concordance between disease activity and functional impairment; NLR and MLR (ρ = 0.78, *p* < 0.001), reflecting the interconnected nature of these inflammatory ratios; CRP and fibrinogen (ρ = 0.66, *p* < 0.001), demonstrating their shared role as acute-phase reactants.

Moderate positive correlations were observed between: Fibrinogen and FPR (ρ = 0.46, *p* < 0.001), NLR and PLR (ρ = 0.51, *p* < 0.001), PLR and MLR (ρ = 0.51, *p* < 0.001), age and glucose (ρ = 0.41, *p* < 0.01), age and BMI (ρ = 0.34, *p* < 0.01), BASDAI and fibrinogen (ρ = 0.39, *p* < 0.01), BASFI and fibrinogen (ρ = 0.32, *p* < 0.05), CRP and NLR (ρ = 0.37, *p* < 0.01), CRP and FPR (ρ = 0.30, *p* < 0.05).

**Figure 2:**
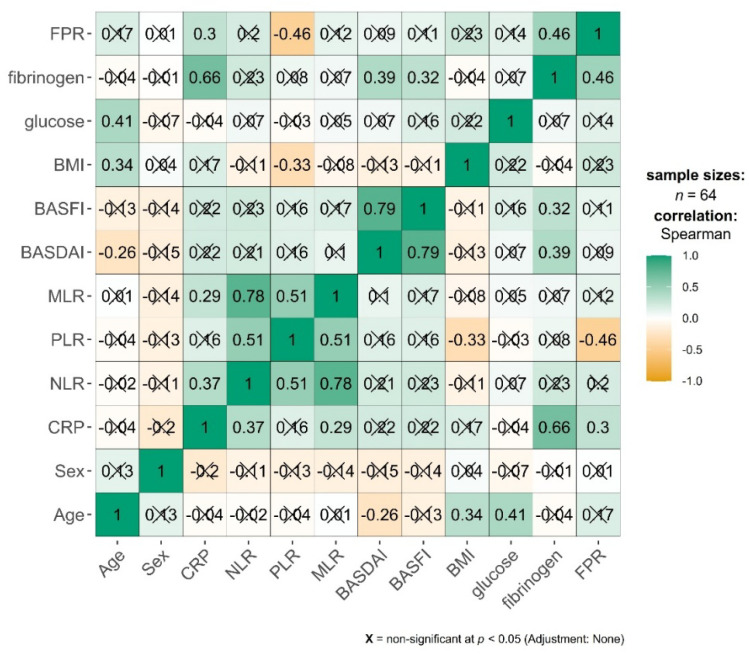
Heatmap with Spearman correlations between demographic, clinical, and laboratory variables. Green represents strong positive correlations (ρ = 1.0), and orange represents strong negative correlations (ρ = −1.0).

Negative correlations of clinical interest included: PLR and FPR (ρ = −0.46, *p* < 0.001), PLR and BMI (ρ = −0.33, *p* = 0.008), age and BASDAI (ρ = −0.26, *p* = 0.035), as shown in Figure 2.

Notably, sex showed no significant correlations with any of the measured variables. Traditional inflammatory markers (CRP, NLR, PLR, MLR) demonstrated weak to moderate intercorrelations but showed limited association with disease activity indices.

### 3.3. Principal Component Analysis of Inflammatory Markers

Principal component analysis with varimax rotation was performed on six inflammatory variables (CRP, NLR, PLR, MLR, fibrinogen, and FPR) to identify underlying patterns of inflammation in the spondylarthritis cohort.

Bartlett’s Test of Sphericity was highly significant (χ^2^ = 186, df = 15, *p* < 0.001), confirming that the correlation matrix differed significantly from an identity matrix and that the variables were sufficiently intercorrelated for PCA. Given the low KMO value, the following PCA results should be regarded as exploratory and interpreted with caution.

The Kaiser–Meyer–Olkin (KMO) measure of sampling adequacy yielded an overall MSA of 0.345, which falls below the commonly recommended threshold of 0.5. Individual variable MSA (Measure of Sampling Adequacy) values ranged from 0.52 (PLR) to 0.648 (CRP), with only CRP achieving a marginally acceptable level. These low KMO values suggest that the sample size (*n* = 64) may be insufficient relative to the number of variables analyzed, or that the variables share limited common variance suitable for factor extraction. Despite this limitation, the analysis was pursued given the study’s exploratory nature and the theoretical relevance of these inflammatory markers.

**Figure 3:**
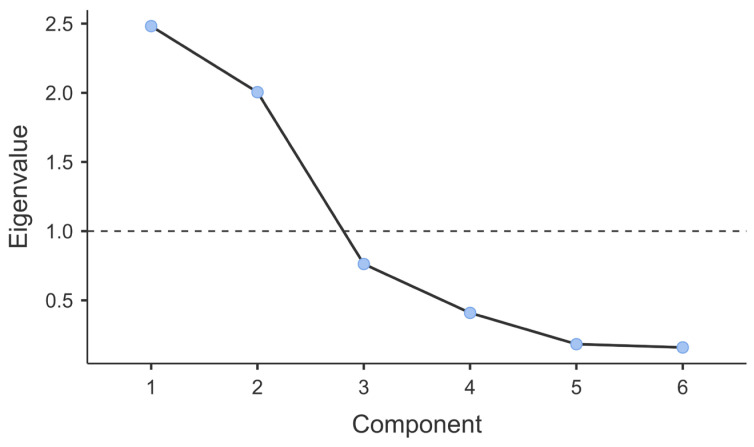
Scree plot of the Principal Component Analysis of inflammatory markers.

Examination of the scree plot (Figure 3) revealed a clear elbow after the second component, supporting retention of two components. Initial eigenvalues for the first two components were 2.48 and 2.00, respectively, all exceeding the Kaiser criterion (eigenvalue >1.0). Components 3–6 had eigenvalues below 1.0, indicating they contributed minimal unique variance.

The two retained components collectively explained 74.8% of the total variance in inflammatory markers (Component 1: 41.4%, Component 2: 33.41%). The varimax rotation successfully achieved orthogonality, with inter-component correlations of 0.00, confirming that the two components represent independent inflammatory dimensions.

Component 1 (41.36% of variance) was characterized by strong positive loadings from NLR (0.897), PLR (0.834), and MLR (0.830). This component represents leukocyte-derived inflammatory ratios, reflecting interconnected neutrophil-, platelet-, and monocyte-mediated inflammation, although this interpretation remains exploratory.

Component 2 (33.41% of variance) showed high loadings for classical acute-phase reactants, CRP (0.774), FPR (0.831), and fibrinogen (0.883). This component reflects the acute-phase/fibrinogen response. Notably, FPR clustered with classical acute-phase reactants rather than with leukocyte-derived ratios, suggesting that FPR predominantly captures systemic acute-phase inflammatory burden.

Uniqueness values revealed that NLR (0.152) and fibrinogen (0.206) were most comprehensively explained by the component structure, while CRP (0.348) and MLR (0.289) retained more unique variance not captured by the two components. FPR showed a uniqueness of 0.264, indicating that approximately 74% of its variance was accounted for by the two-component solution.

### 3.4. Comparison of Clinical Characteristics by HLA-B27

Table 2 presents the comparison of demographic, clinical, and laboratory characteristics between HLA-B27-positive (*n* = 7, 10.9%) and HLA-B27-negative (*n* = 57, 89.1%) patients.

Inflammatory markers, including CRP (*p* = 0.846), NLR (*p* = 0.763), PLR (*p* = 0.111), and MLR (*p* = 0.426), showed no significant between-group differences, despite numerical differences. Disease activity and functional indices were comparable, with BASDAI (*p* = 0.395) and BASFI (*p* = 0.957) showing similar values between HLA-B27-positive and HLA-B27-negative patients, although HLA-B27-positive patients demonstrated numerically higher median BASDAI scores (6.2 vs. 5.0).

Fibrinogen levels (*p* = 0.533) and the fibrinogen-to-platelet ratio (*p* = 0.220) did not differ significantly between groups, though HLA-B27 positive patients showed numerically higher values for both parameters (median fibrinogen: 379 vs. 385 mg/dL; median FPR: 1.64 vs. 1.35).

### 3.5. ROC Curve Analysis to Evaluate Inflammatory Markers as Predictors of Disease Activity

**Figure 4:**
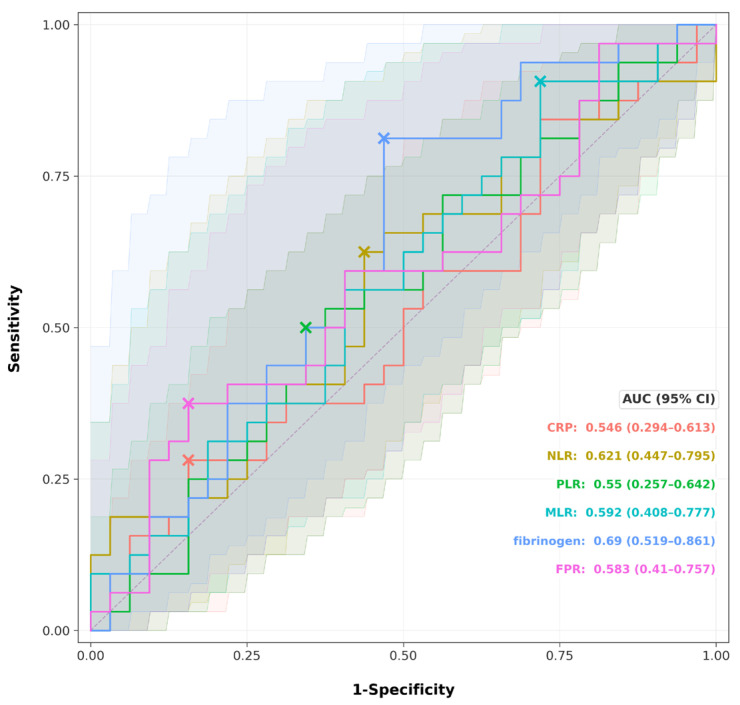
ROC curves with 95% Confidence Intervals with inflammatory markers predicting disease activity (BASDAI ≥ 4). Each colored line represents the ROC curve for a specific marker: CRP (red), NLR (yellow-green), PLR (green), MLR (cyan), fibrinogen (blue), and FPR (pink). The × symbol on each curve indicates the optimal cutoff point determined by the Youden index (maximum sensitivity + specificity − 1). The shaded areas surrounding each curve represent the 95% confidence intervals for the corresponding AUC, generated by bootstrap resampling. The dashed diagonal line represents the reference line of no discrimination (AUC = 0.5). AUC values with 95% CI are displayed in the bottom-right corner. Among all markers, fibrinogen demonstrated the highest discriminative ability (AUC = 0.69, 95% CI: 0.519–0.861), and was the only marker whose confidence interval did not cross the 0.5 threshold.

ROC curve analysis revealed that fibrinogen demonstrated the highest discriminative ability for high disease activity (BASDAI ≥ 4), with an AUC of 0.690 (95% CI: 0.519–0.861, *p* < 0.001, optimal cutoff: 325 mg/dL), as shown in Figure 4. NLR showed the second-highest AUC at 0.621 (95% CI: 0.447–0.795, optimal cutoff: 3.16), followed by MLR (AUC = 0.592, 95% CI: 0.408–0.777, optimal cutoff: 0.268), FPR (AUC = 0.583, 95% CI: 0.410–0.757, optimal cutoff: 1.444), PLR (AUC = 0.550, 95% CI: 0.257–0.642, optimal cutoff: 109.5), and CRP (AUC = 0.546, 95% CI: 0.294–0.613, optimal cutoff: 10.6 mg/dL). Among all markers, only fibrinogen’s 95% confidence interval did not cross the 0.5 threshold, indicating marginal but statistically significant discriminative ability. While FPR successfully discriminated SA subtypes (*p* = 0.003), its modest AUC for disease activity (0.583) reinforces its primary role as a phenotypic trait marker rather than a state marker of symptom severity.

**Figure 5:**
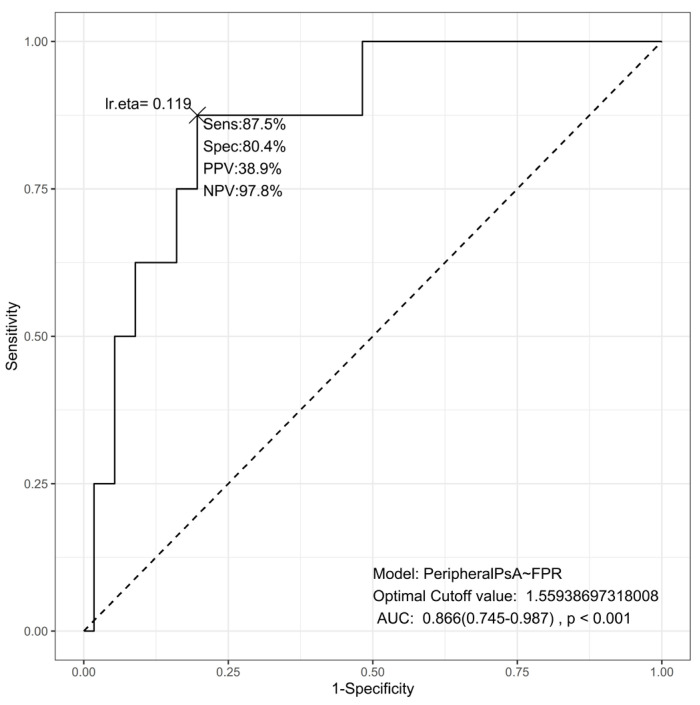
ROC Curve with FPR predicting peripheral spondyloarthritis.

Additionally, a separate ROC analysis was performed to evaluate FPR as a discriminator between peripheral SA (*n* = 8) and non-peripheral SA (axial + psoriatic SA combined, *n* = 56), as shown in Figure 5. FPR demonstrated excellent discriminative ability, with an AUC of 0.866 (95% CI: 0.745–0.987, *p* < 0.001). The optimal FPR cutoff derived from the Youden index was 1.559, yielding a sensitivity of 87.5%, a specificity of 80.4%, a positive predictive value (PPV) of 38.9%, and a negative predictive value (NPV) of 97.8%. The high NPV indicates that an FPR value below 1.559 virtually rules out peripheral SA, while the modest PPV reflects the low prevalence of peripheral SA in the study cohort. These findings support FPR as a robust phenotypic biomarker for identifying peripheral SA, in contrast to its modest performance in predicting disease activity.

## 4. Discussion

The present study investigated the discriminative and predictive utility of systemic hematologic inflammatory markers in a heterogeneous cohort of patients with spondyloarthritis. Our findings reveal a significant biomarker gap: while specific markers—particularly the novel FPR—can effectively differentiate between SA subtypes, they fail to track the subjective disease activity reported by patients.

The most striking finding in our analysis is the FPR’s capacity to distinguish among SA subtypes, particularly to identify the peripheral SA phenotype. Patients with peripheral SA exhibited significantly higher FPR (median 1.88) compared to those with axial SA (median 1.33) and psoriatic SA (median 1.36). This suggests that FPR may function as a “trait marker” rather than a “state marker”.

The elevated FPR in peripheral SA, combined with the patients’ significantly younger age (median age 42.5 years), suggests a distinct neutrophil-hypercoagulable inflammatory profile early in the disease course. This observation is supported by our PCA, which showed that FPR clustered with CRP and fibrinogen on the “acute-phase/fibrinogen axis” (Component 2, 33.4% of variance), rather than with leukocyte-derived ratios.

Although conventional hematologic ratios such as NLR and PLR have shown inconsistent associations with disease activity in SA, our findings suggest that the fibrinogen-to-platelet ratio (FPR) may have phenotypic discriminatory value, particularly for identifying peripheral SA [12]. This difference likely reflects underlying pathophysiologic heterogeneity between axial and peripheral disease subsets. Axial SA is predominantly characterized by compartmentalized inflammation at the enthesis and sacroiliac joints, largely driven by the IL-17/IL-23 axis, and is frequently accompanied by normal or only mildly elevated acute-phase reactants [1,13]. In contrast, peripheral SA often exhibits more overt synovial inflammation with greater systemic inflammatory spillover, including stronger IL-6–mediated hepatic acute-phase responses. Because fibrinogen is a sensitive IL-6–dependent acute-phase protein and platelets respond to inflammatory cytokine signaling, FPR may better capture this systemic inflammatory signature. Therefore, rather than reflecting overall disease activity, FPR may function as a marker of inflammatory phenotype, distinguishing peripheral from predominantly axial presentations. This interpretation aligns with the concept that SA subtypes differ not only clinically but also in their systemic inflammatory expression, which may not be adequately captured by leukocyte-derived indices such as NLR or PLR [14].

The phenotypic discriminatory value of FPR was further confirmed by the dedicated ROC analysis for peripheral SA identification. FPR demonstrated excellent discriminative ability (AUC = 0.866, 95% CI: 0.745–0.987, *p* < 0.001), with an optimal cutoff of 1.559 yielding 87.5% sensitivity and 80.4% specificity. The remarkably high negative predictive value (97.8%) suggests that FPR values below this threshold can reliably exclude peripheral SA, which may be clinically useful in the early diagnostic workup. The lower positive predictive value (38.9%) is expected, given the low prevalence of peripheral SA in this cohort (12.5%), and does not diminish FPR’s utility as a screening or stratification tool. This strong phenotypic discriminative performance (AUC = 0.866) stands in marked contrast to FPR’s modest predictive ability for disease activity (AUC = 0.583 for BASDAI ≥ 4), further reinforcing the distinction between trait and state biomarkers. Future studies should evaluate whether the proposed FPR cutoff of 1.559 retains its discriminative performance in independent, larger cohorts and whether it can contribute to earlier phenotypic classification in clinical practice.

A critical challenge in rheumatology is the frequent mismatch between objective inflammatory burden and patient-reported outcomes [15,16,17,18]. Our ROC analysis confirmed this dissociation: despite established correlations between markers like CRP, NLR, and fibrinogen, none could accurately predict a high disease activity state (BASDAI ≥ 4). Although the corrected analysis showed that fibrinogen achieved the highest AUC (0.690, 95% CI: 0.519–0.861) and was the only marker whose confidence interval did not cross 0.5, overall discriminative performance remained modest. The remaining markers, including FPR (AUC = 0.583), NLR (AUC = 0.621), and MLR (AUC = 0.592), had confidence intervals that crossed 0.5. This “modest discriminative ability” suggests that the BASDAI, while essential for clinical management, captures a multidimensional experience of pain and fatigue that is not solely driven by systemic inflammation. This highlights the need for clinicians to differentiate between inflammatory disease activity and symptom severity, which may be influenced by age, BMI, or metabolic factors [19,20].

The use of PCA allowed us to move beyond single-marker analysis to view the “inflammatory architecture” of SA. By identifying two independent dimensions— a leukocyte-derived inflammatory axis (NLR, PLR, MLR) and an acute-phase/fibrinogen axis (CRP, fibrinogen, FPR)—we have shown that systemic inflammation in SA is not a monolithic process [21]. This level of explained variance (74.8%) suggests that a single marker, such as CRP, is insufficient to characterize the total inflammatory burden. Notably, FPR clustered exclusively with acute-phase reactants (Component 2), rather than with leukocyte-derived ratios, reinforcing its biological link to IL-6–dependent hepatic acute-phase activation rather than to peripheral leukocyte dynamics. Although fibrinogen is involved in both the inflammatory cascade and coagulation pathways, the absence of direct coagulation markers (e.g., D-dimer, thrombin generation assays) in this study precludes definitive characterization of the identified components as reflecting a hypercoagulable state. Future biomarker strategies should likely focus on “multi-marker panels” that span these distinct pathways to better capture the biological complexity of the disease [22,23].

This study is limited by its cross-sectional design and the relatively small sample size, particularly of the peripheral SA subgroup, which may affect the robustness of between-group comparisons and increase variability in the estimates. Although nonparametric statistical methods were employed to account for non-normal distributions and small group sizes, the statistical power to detect differences in the peripheral SA group remains limited. The observed differences in FPR and fibrinogen, while statistically significant after correction for multiple comparisons, should be interpreted as hypothesis-generating findings that require confirmation in larger, adequately powered cohorts. Furthermore, the unequal distribution of patients across SA subtypes reflects the natural epidemiological variation in clinical practice but nonetheless limits the generalizability of subgroup-specific conclusions. The PCA was conducted as an exploratory analysis to identify potential patterns of co-variation among inflammatory markers. Given the suboptimal KMO value, these findings should be considered hypothesis-generating and require validation in larger datasets. The absence of comprehensive comorbidity data, including cardiovascular disease, metabolic syndrome, and smoking status, limits the generalizability of the findings. Nonetheless, our results demonstrate that while traditional and novel markers cannot yet replace patient-reported indices for tracking day-to-day symptom fluctuations, they remain invaluable for phenotypic differentiation. Specifically, the FPR emerges as a robust tool for identifying the distinct hypercoagulable inflammatory state characteristic of peripheral spondyloarthritis.

## 5. Conclusions

In conclusion, this study demonstrates that systemic hematologic inflammatory markers serve distinct and complementary roles in spondyloarthritis, supporting a clear distinction between phenotypic and activity biomarkers. FPR emerged as a robust phenotypic biomarker with excellent discriminative ability for identifying peripheral SA and a remarkably high negative predictive value, suggesting its potential utility as a screening tool in the early diagnostic workup. In contrast, FPR showed only modest capacity to predict patient-reported disease activity, confirming that it functions as a trait marker of inflammatory phenotype rather than a state marker of symptom severity. Exploratory PCA revealed that systemic inflammation in SA is not a monolithic process but comprises at least two independent dimensions: a leukocyte-derived inflammatory pathway and an acute-phase/fibrinogen pathway. Notably, FPR clustered exclusively with acute-phase reactants rather than with leukocyte-derived ratios, reinforcing its biological link to IL-6–dependent systemic inflammation and distinguishing it mechanistically from conventional hematologic indices such as NLR and PLR. Among all markers evaluated for disease activity prediction, fibrinogen demonstrated the highest discriminative performance, although the overall predictive ability of all hematologic markers remained modest, highlighting the multidimensional nature of patient-reported outcomes that extends beyond measurable systemic inflammation. These findings should be interpreted in light of several limitations, including the cross-sectional design, the small sample size with a notably limited peripheral SA subgroup, the suboptimal sampling adequacy for PCA, and the absence of direct cytokine, coagulation, and comprehensive comorbidity data. Further prospective studies with larger, well-balanced cohorts are warranted to validate the proposed FPR cutoff for peripheral SA identification, to confirm the two-dimensional inflammatory architecture, and to investigate the underlying pathophysiological mechanisms through direct cytokine profiling.

## Figures and Tables

**Table 1:** Characteristics of the patients.

Characteristics	Total (*n* = 64)	Axial SA (*n* = 32)	Peripheral SA (*n* = 8)	Psoriatic SA (*n* = 24)	*p*-Value
Age, years					<0.001
mean ± SD	53.5 ± 12.8	50.1 ± 9.4	41.5 ± 13.4	62 ± 11.5	
median (IQR)	53 (43.8–62.3)	47.5 (43–55.3)	42.5 (37–45.8)	59.5 (53–70.5)	
Sex, male (yes, %)	28 (43.8%)	20 (31.2%)	2 (3.1%)	6 (9.4%)	0.010
BMI, kg/m^2^					0.113
mean ± SD	27 ± 5.94	25.9 ± 5.79	25.3 ± 5.59	29 ± 5.92	
median (IQR)	26.6 (22.3–32)	25.1 (22–28.2)	25.1 (22.5–26.6)	30.3 (25.5–32.1)	
Diabetes, yes (*n*, %)	7 (10.6%)	3 (4.7%)	1 (1.6%)	3 (4.7%)	<0.012
HLAB27, positive (*n*, %)	7 (10.9%)	4 (6.3%)	3 (4.7%)	0	<0.001
CRP, mg/dL					0.75
mean ± SD	11.9 ± 17.3	11.4 ± 16.1	14.2 ± 19.7	11.8 ± 18.9	
median (IQR)	5.62 (1.62–11.3)	5.68 (1.27–14)	9.29 (2.78–12.3)	4.8 (1.86–9.55)	
NLR					0.474
mean ± SD	11.4 ± 68.5	2.66 ± 1.23	2.94 ± 1.13	25.8 ± 112	
median (IQR)	2.58 (1.72–3.52)	2.35 (1.53–3.52)	2.72 (2.14–3.47)	2.55 (1.92–3.72)	
PLR					0.374
mean ± SD	158 ± 65.4	159 ± 60.1	128 ± 41.3	166 ± 77.1	
median (IQR)	151 (114–189)	168 (104–204)	134 (105–160)	1419 (122–1849)	
MLR					0.764
mean ± SD	1.28 ± 0.14	0.26 ± 0.11	0.28 ± 0.1	0.32 ± 0.18	
median (IQR)	0.27 (0.2–0.35)	0.26 (0.18–0.34)	0.28 (1.2–0.35)	0.26 (0.21–0.36)	
BASDAI					0.247
mean ± SD	5.26 ± 1.44	5.5 ± 1.65	5.64 ± 1.24	4.81 ± 1.12	
median (IQR)	5 (4.07–6.23)	5 (4.9–6.75)	5.7 (4.9–6.05)	4.6 (4–5.62)	
BASFI					0.233
mean ± SD	4.91 ± 1.57	5.16 ± 1.71	5.34 ± 1.82	4.43 ± 1.19	
median (IQR)	4.2 (3.7–6)	4.3 (3.7–6.7)	4.9 (4.52–6.05)	4 (3.68–4.82)	
Fibrinogen					0.037 * 1 vs. 2: 0.04 1 vs. 3: 0.71 2 vs. 3: 0.037
mean ± SD	404 ± 117	392 ± 105	496 ± 116	390 ± 123	
median (IQR)	385 (323–455)	378 (324–456)	452 (439–522)	361 (312–427)	
Platelets					0.381
mean ± SD	300 ± 87.5	159 ± 60.1	128 ± 41.3	148 ± 46.7	
median (IQR)	271 (243–337)	290 (244–412)	261 (222–314)	259 (246–305)	
FPR					0.003 * 1 vs. 2: 0.002 1 vs. 3: 0.21 2 vs. 3: 0.03
mean ± SD	1.41 ± 0.44	1.28 ± 0.3	1.91 ± 0.4	1.42 ± 0.49	
median (IQR)	1.36 (1.20–1.57)	1.33 (1.09–1.44)	1.88 (1.62–2.21)	1.32 (1.22–1.51)	

SA, spondyloarthritis; CRP, C-reactive protein; NLR, Neutrophil-to-Lymphocyte Ratio; PLR, Platelet-to-Lymphocyte Ratio; MLR, Monocyte-to-Lymphocyte Ratio; BASDAI, Bath Ankylosing Spondylitis Disease Activity Index; BASFI, Bath Ankylosing Spondylitis Disease Functional Index; FPR, Fibrinogen-to-Platelet Ratio; *, statistically significative.

**Table 2:** Characteristics of patients stratified by HLA-B27 status.

Characteristics	Total (*n* = 64)	Axial SA (*n* = 32)	Peripheral SA (*n* = 8)	*p*-Value
Age, years				0.067
mean ± SD	53.5 ± 12.8	54.6 ± 12.3	44.6 ± 13.9	
median (IQR)	53 (43.8–62.3)	53 (45–63)	42 (38.5–53.5)	
Sex, male (yes, %)	28 (43.8%)	27 (42.2%)	1 (1.6%)	0.125
BMI, kg/m^2^				0.613
mean ± SD	27 ± 5.94	27.1 ± 5.99	25.8 ± 5.86	
median (IQR)	26.6 (22.3–32)	27 (22.3–32)	26.5 (21.3–29.5)	
Diabetes, yes (*n*, %)	7 (10.6%)	7 (10.9%)	0	0.326
Spondylarthritis subtypes (yes, %)				0.012 *
Axial	32 (50%)	28 (43.8%)	4 (6.3%)	
Peripheral	8 (12.5%)	5 (7.8%)	3 (4.7%)	
Psoriatic	24 (37.5%)	24 (37.5%)	0	
CRP, mg/L				0.846
mean ± SD	11.9 ± 17.3	11.7 ± 17	13.7 ± 21.6	
median (IQR)	5.62 (1.62–11.3)	5.76 (1.54–11.3)	5.47 (2.28–11.9)	
NLR				0.763
mean ± SD	11.4 ± 68.5	12.4 ± 7.26	2.51 ± 0.84	
median (IQR)	2.58 (1.72–3.52)	2.55 (1.71–3.54)	2.61 (2.07–3.03)	
PLR				0.111
mean ± SD	151 ± 53.6	54.1 ± 0.98	40.4 ± 0.97	
median (IQR)	150 (112–188)	151 (114–191)	117 (102–139)	
MLR				0.426
mean ± SD	1.28 ± 0.14	0.29 ± 0.15	0.24 ± 0.08	
median (IQR)	0.27 (0.2–0.35)	0.28 (0.2–0.36)	0.24 (0.21–0.28)	
BASDAI				0.395
mean ± SD	5.26 ± 1.44	5.19 ± 1.41	5.79 ± 1.69	
median (IQR)	5 (4.07–6.23)	5 (4.10–6)	6.2 (4.3–6.95)	
BASFI				0.957
mean ± SD	4.91 ± 1.57	4.88 ± 1.5	5.19 ± 2.18	
median (IQR)	4.2 (3.7–6)	4.2 (3.7–6)	4.3 (3.6–6.4)	
Fibrinogen				0.533
mean ± SD	404 ± 117	399 ± 112	451 ± 156	
median (IQR)	385 (323–455)	385 (321–452)	379 (349–534)	
FPR				0.220
mean ± SD	1.5 ± 0.78	1.49 ± 0.81	1.63 ± 0.38	
median (IQR)	1.36 (1.22–1.62)	1.35 (1.14–1.56)	1.64 (1.3–1.89)	

*, statistically significative.

## Data Availability

The original data presented in the study are openly available in Zenodo (version v1.0.) at DOI https://doi.org/10.5281/zenodo.18763989.

## Abbreviations

The following abbreviations are used in this manuscript:

CRP	C-reactive protein
ESR	erythrocyte sedimentation rate
FPR	fibrinogen-to-platelet ratio
MLR	monocyte-to-lymphocyte ratio
NLR	neutrophil-to-lymphocyte ratio
PLR	platelet-to-lymphocyte ratio
SII	systemic immune-inflammation index
SA	Spondyloarthritis
